# Exploring Dendroflora Diversity and Ecology in an Urban Arboretum from Western Romania: The Role of Plant Life-Form and Plant Family in Urban Woody Phytocoenosis

**DOI:** 10.3390/plants14050717

**Published:** 2025-02-26

**Authors:** Madalina Iordache, Laurentiu Zamfir, Alexandra Becherescu, Ana Codruţa Chiş

**Affiliations:** Department of Sustainable Development and Environmental Engineering, Faculty of Agriculture, University of Life Sciences “King Mihai I”, 300645 Timisoara, Romania; laurentiuzamfir55@gmail.com (L.Z.); alexandrabecherescu@usvt.ro (A.B.); codruta_chis@usvt.ro (A.C.C.)

**Keywords:** bioform, chorology, phytogeographic, element, geoelement, moisture, temperature, soil pH

## Abstract

The dendroflora of an urban arboretum (The Botanic Park of Timișoara, Romania), consisting of 193 species, was ecologically characterized as bioforms, phytogeographical elements, and preferences for moisture, temperature, and soil pH. The aim of the research was to determine whether the native ecological requirements of the woody species, along with certain biological and evolutionary traits of them, such as plant life-form and plant family, could serve as tools for explaining and understanding the strategies employed by the urban woody phytocoenoses to acclimate and adapt to an established environment, such as an urban arboretum. The inventoried species are grouped in 111 genera and 45 families. The native and non-native dendroflora share 16 common families. The most representative family both in the native and non-native dendroflora is *Rosaceae*. The monotypic families are largely present (22.22% in the native dendroflora, and 42.22% in the non-native dendroflora). The plant life-form spectrum is dominated by megaphanerophytes (49%), followed by mesophanerophytes (41%). The chorological spectrum of the native species comprises 16 chorological types and is dominated by Eurasians (32%) and Europeans (30%). The species characteristics of the Pontic-Carpathian space, to which Romania belongs, are rare in the analyzed urban botanical park (4%). The mesophyte, mesothermal and slightly acido-neutrophilous species dominate both the native and non-native dendroflora. In the acclimation process of the non-native dendroflora, 37% of species exceeded their native requirements for moisture, 41% for temperature, and 50% for soil pH. The species requirements for temperature are associated to those for moisture and soil pH. The results show the potential of the analysed woody species to exceed their native requirements within the acclimation and adaption process, and in this process, for the studied temperate site, the plant life-form is important, and also the plant family. The species of the analyzed urban arboretum, both native and non-native, are taxonomically and biogeographically diverse, with specific habitat requirements, suggesting their great ability in acclimating, adapting and resisting.

## 1. Introduction

The preoccupation of studying the urban green spaces has increased in the last decades since the scientist forecasts announce the expansion of urban settlements as the main living areas of the human population. Among the diversity of the approached subjects, the ecosystem services provided by the urban green spaces are by large interest (improvement of the quality of the environmental factors, sanogenesis, carbon sequestration, climatic buffer, aesthetic benefits). The biodiversity services brought by the urban green spaces are of interest for humanity because its survival is linked to the urban sustainability as safe and reliable living environment. The new approaches regarding the design and the management of the urban green spaces are in present established, or these should be, considering the biodiversity strategy, enhancing the role of the urban green spaces as biodiversity hotspots in the urban environment.

In the urban planning of the green spaces, the species composition is a factor in rising up the potential of these areas to fulfill their roles in the sustainability of the urban environment. But, most of the time, when urban parks are established, other more pragmatic factors take precedence, such as the human investment for their long-term maintenance or the receptiveness and satisfaction of citizens with the design offered by the selected species. The settlement of the urban tree parks is an option for keeping the sustainability of cities for several well known important reasons such as higher resistance to pollution stress, efficient rates of carbon sequestration, but also for other less mediated roles like seasonal indicators due to their successive life cycles, urban identity providers [1], or drivers of the biogeochemical cycles.

The fulfillment of the ecosystem services by the urban green spaces represents the motivation for establishing these parks, and the efficiency of this objective is reflected in the selection of plant categories used for their settlement, so that the ratio between woody plants and herbaceous plants becomes important, as woody plants offer several advantages over herbaceous ones when the efficiency of ecosystem services provided by the urban green spaces are analyzed. The tree size and implicitly the biomass amount, both the underground and the above ground one, the foliar surface of the canopy represent advantages regarding the quantity of the sequestrated carbon or drought resistance [2]. In the temperate areas, the establishment of the urban green spaces with tree-dominated species composition, exploits a proven tree strategy [3] to develop height-mediated hydraulic mechanisms to fight against the freeze-thaw stress and sustain their long-term survival despite unfavorable periods. The long life span of the trees as compared to the herbaceous plants is an opportunity for a long time carbon sequestration through the above ground biomass [4], regulating thus the local carbon cycle and acting like long lasting carbon sinks. The herbaceous plants are more vulnerable to the urban stress factors which, associated to their short life cycles make them less desirable for reaching environmental gains in the urban areas. Previous studies showed that, the short life cycle of the annual weeds in several urban green spaces in addition to moisture deficit and drought climate favored their presence only in the favorable seasons [5], which make them unreliable on long time for the urban environmental sustainability. The resilience of the urban ecosystems to the various environmental threats (droughts, air pollution, freeze, soil leaching, floods) is enhanced and supported by the urban woody vegetation and is increasing with the species diversity [6]. The ecosystem services provided by the urban green spaces are the main goal of the urban sustainability and for its achievement the ratio between the woody and herbaceous plants in the urban parks is considered. The urban green spaces remain the main connection between the continuously growing urbanization areas and the natural environment, and their establishment represents a mean of mitigating specific effects of urbanization like natural habitat disruption, soil sealing and defective infiltration precipitations, species erosion.

The urbanization phenomenon is responsible for certain ecological disturbances in the urban biocoenoses, such as the biological invasion [7] or decrease of species richness in cities. A study on urban remnant patches of forest after urban surrounding showed that the intensity of urban expansion is a factor which decreases the woody species richness in this type of urban green space [8]. Therefore, the species selection in establishing of the urban green spaces remains the suitable option to increase the urban diversity and to meet its goals in providing urban resilience and sustainability.The climate changes faced by human-inhabited urban environments need, in order to be countered, urban management measures that include green infrastructure strategies. The warnings of scientists regarding the phenomenon of global warming, fully felt in cities, should determine a proactive approach towards the measures that could be taken to reduce this phenomenon, and in this sense, the presence of trees in cities, in the form of arboretums or as street alignments, could be an exploitable solution.

The aim of the research was to identify whether there are associations between the native ecological requirements of the woody species forming the arboretum, and also between these and certain biological and evolutionary traits of them, such as plant life-form and plant family, as ways to describe the potential of the identified species to keep or shift their native ecological requirements in developing adapting relationships to the urban environmental factors.

## 2. Results and Discussion

The dendroflora of the Botanic Park of Timișoara consists of 193 species: 73 species are native to Romania (Table 1) and 120 species are non-native species (Table 2), grouped in 111 genera and 45 families. This species richness is high and comparable to other urban woody parks in the world [9,10], but the species richness in the urban parks should be assessed also depending on the site size, its geographical characteristics or management preferences [11]. Predominant are the non-native species which represent 62.17% from the total dendroflora and the angiosperms which represent 84.46% as compared with the gymnosperms (15.54%). Seven native species belong to two gymnosperm families (*Pinaceae* and *Taxaceae*), and 23 non-native species belong to five gymnosperm families (*Cupressaceae*, *Ginkgoaceae*, *Pinaceae*, *Taxodiaceae*, and *Taxaceae*) (Table 1 and Table 2).

The dominance of the non-native dendroflora in the studied park is consistent with other findings regarding the urban dendroflora from abroad, showing the general tendency in the urban green areas to be dominated by the presence of the alien or exotic species [12,13], as a consequence of the aesthetic and ornamental motivations or due to the lack of scientifically based planning among authority decisional factors since the recommendations indicate the settlement of the urban arboreta based on the native species which are more susceptible to comply with the local ecological criteria [14] to avoid the ecological unbalances (such as alien species invasion with all its associated consequences) and other problems related to population health (like allergenic potential of the pollen). However, the non-native species contribute more to the phylogenetic diversity in the urban green spaces [13]. But, several studies encourage nowadays the native tree species to be part of the urban dendroflora, sustaining their findings through a better plasticity of the native species in facing the local environmental stressors, a better long-time resilience, and a better ability to support the rest of the local native biodiversity [15].

The native and alien dendroflora of the studied arboretum share 16 common families. The most representative family both in the native and alien dendroflora is *Rosaceae*, which is represented by 14 species (19%) in the native dendroflora and respectively by 20 species (10%) in the non-native dendroflora. The family *Rosaceae* has been also found to be the most representative in other studies regarding the biodiversity and ecological succession of the urban green spaces [16,17]. The predominance of the family *Rosaceae* was found by Lakicevic et al. (2022) to be a characteristic of the dendroflora from the temperate climatic regions, where Romania belongs [18]. This aspect has been previously observed by Postarnak and Zhavoronkov (2023) as a characteristic of the urban flora of other cities. The monotypic families are largely present in the dendroflora of the analyzed urban park, namely 10 families in the native dendroflora (22.22% of total) and 19 families (42.22% of total) in the non-native dendroflora [17].

The dominance of the non-native species (62.17%) in the analyzed urban park, combined with the dominance of monotypic families, are characteristics of the urban green spaces and a strategy of the urbanistic managements to increase the species richness in the urban parks, because the species richness is an indicator of their sustainability. However, despite this desirable goal, the voluntarily introduced non-native species could gradually transform into invasive species, threatening the local diversity and sometimes compromising the adjacent ecosystems [19]. A limiting factor for this inconvenient is the urban environment itself, because it offers limited space for spontaneously emerging since the great part of the urban soil is sealed and controlled. But this aspect increases the urban plant competition for the safe sites [7] defined as appropriate sites for germination and emergence, and within this competition the herbaceous plants are more likely to succeed [7]. Thus, the urban parks, voluntarily settled by man, remain the major way to have woody plants in cities to benefit from their environmental advantages. The dominant non-native species in the park dendroflora has been investigated in the present study only for the plant life-forms and ecological requirements for moisture, temperature and soil pH, but the implications of the dominance of the alien woody species in urban space could have also other implications. For example, although the presence of the native woody species in the urban green spaces has been found to favour the relationships between flora and fauna, supporting or enhancing the local biodiversity, there are also studies regarding diverse non-native woody taxa in urban parks which showed that the woody non-native species impoverish these relationships and lower the diversity of the native communities [20]. Some studies highlighted the role of the trees as ecological indicators in the urban green planning, recommending the prioritization of the native species in urban planting [21] to keep on the urban identity. But others suggested that the urban allochthonous woody species have generative potential to be used as seed banks for the urban greenery [22].

**Figure 1:**
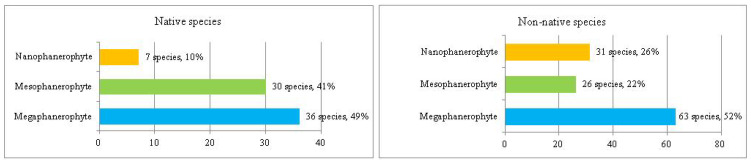
Spectrum of plant life-forms in the native and non-native dendroflora of the Botanic Park from Timișoara City.

The analysis of the plant life-form spectrum of the park dendroflora showed that the main plant life-forms are megaphanerophytes (49%), followed by mesophanerophytes (41%) (Figure 1).

The megaphanerophytes dominate both the native (49%) and the non-native dendroflora (52%), but the native mesophanerophytes are almost double as species number than the non-native mesophanerophytes. The nanophanerophytes are low represented in the native dendroflora (10%) but are more representative in the non-native one (26%). In all types of ecosystems, in the competition for resources, the ratio between phanerophytes and other plant life-forms is important, including the ratio between megaphanerophytes and mesophanerophytes. In the natural ecosystems, the ratio between these two categories of plants provides information about vegetation dynamics and about the availability of certain resources (light, water, temperature), which in turn determines specific types of ecological interactions between different species that influence the structure and dynamics of biological communities (competition, parasitism, predation, extinction, ecological succession etc.). For example, a high ratio of megaphanerophytes may indicate an ecosystem dominated by mature forests, an ecosystem that tends toward or has already reached a climax stage, with a developed canopy level, a condition for high biodiversity at this level (habitat for other species, such as birds and insects, support for epiphytic species). A higher ratio of mesophanerophytes may suggest a younger successional stage or an ecosystem where disturbance conditions are present. These are ecosystem functions that are desirable to be exploited into the urban environment by establishing arboretums and urban parks with large-sized woody vegetation and which are sustained through studies and researches. A study regarding mountain grasslands facing with the expansion of the phanerophytes showed that these grasslands are more demanding for light and temperature, but the effect is similar with that manifested by the chamaephytes [23]. Other studies showed that the phanerophytes were least affected by extinction when different habitats have been analyzed [24]; in urban sites, this turns in a high resilience of the phanerophytes to the versatile urban environment It seems that the height of the woody plants in the urban park in one of the most important factors (alongside canopy factors, and tree trunk diameter) in shaping microhabitats and competition strategies in the urban parks. In our study, the megaphanerophytes own 49% in the spontaneous dendroflora and respectively 52% in the alien dendroflora. These percentages of tall trees with long lifespan and slow growth in the park show their urban adaption on long term with all the advantages emerging from here. The tall tree species have been found to be more abundant in the urban green settlements [21,25], these are preferred and recommended because of their better adaption to the urbanization: better regeneration rate, better competitiveness in resources exploitation, better resistance to urban stressors. Several studies indicated strong correlation between the tree size and the presence of some taxons of the urban biodiversity [26]. The studied urban park is an anthropic ecosystem, designed as a dendrological park, and the ratio between mega- and mesophanerophytes was predetermined. The fact that mega- and mesophanerophytes dominate the park (90%) as compared to nanophanerophytes determines a certain vertical and horizontal spatial distribution important in describing habitats and microhabitats and thus the potential of this type of vegetation to support various life forms, which was also the argument for establishing the park in this form, namely to increase the urban biodiversity alongside the urban wellness. However, the selection of tree species composition greatly considers aesthetics when urban arboretums are established [27], therefore the ratio between woody plants with various heights expressed as mega-, meso-, and nanophanerophytes in the urban green spaces are an assumed design option, which should be a median between citizen preferences and experts recommendations. The ratio herbaceous/shrubs/woody plants is not only an issue of urban architecture or aesthetics, but is also a basis in establishing the expectations regarding the ecosystem services provided by the urban green spaces. For example, the tree size is correlated to the CO_2_ stoking capacity in the urban parks [11] and with the capacity to resist to freeze-thaw cycles, because the high trees have bio-physiological (conductive vessels) and physical (hydraulic features) characteristics [3] adapted to this purpose, so that the ecosystem services will not be stopped due to tree dieback. The settlement of compounding species in the urban green spaces should take into consideration the findings of several studies which showed a direct correlation between the anthropogenic built structures around the green space and the biodiversity loss [8], or factors like the species lifespan [1], its efficiency or sensitivity in urban de-pollution [28], or the resilience in front of urbanization expansion, which confer several advantages to the woody vegetation to be chosen. The dominance of a certain plant life-form within a site has been found to be an important biomarker for a certain resource, like hemicryptophytes have been reported for the water availability in several urban habitats [29]. The settlement of the analyzed urban park as a dendrological/arbuscular park aims to harness all the advantages provided by the very presence of the phanerophytes that constitute it: to mitigate certain undesirable environmental phenomena like air pollution, noise pollution, overheating [30], and to bring aesthetic benefits to the urban landscape.

**Figure 2:**
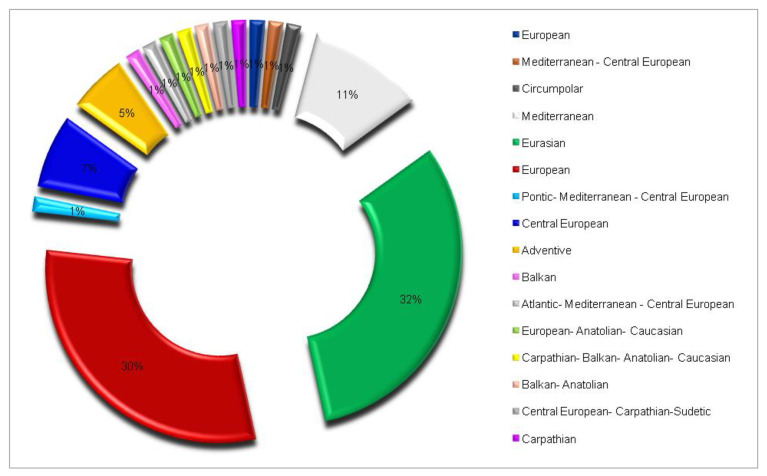
Chorological spectrum regarding the geographic origin of the native woody species in the Botanic Park from Timișoara City.

From chorological point of view, the most numerous native woody species are Eurasian (32%) and European (30%), which shows the dominating European autochthony of the native dendroflora in the studied urban park. The species characteristic of the Pontic-Carpathian space, to which Romania belongs, are rare in the analyzed urban park (4%). There was noticed the high heterogeneity of the phytogeographic elements in the group of the native dendroflora represented by 16 chorological types (Figure 2).

**Figure 3:**
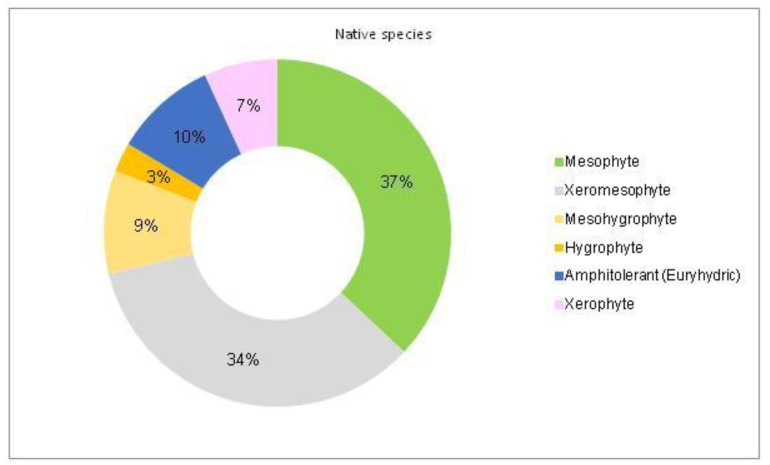
The spectrum of species requirements for moisture in the native dendroflora of the Botanic Park from Timișoara City.

**Figure 4:**
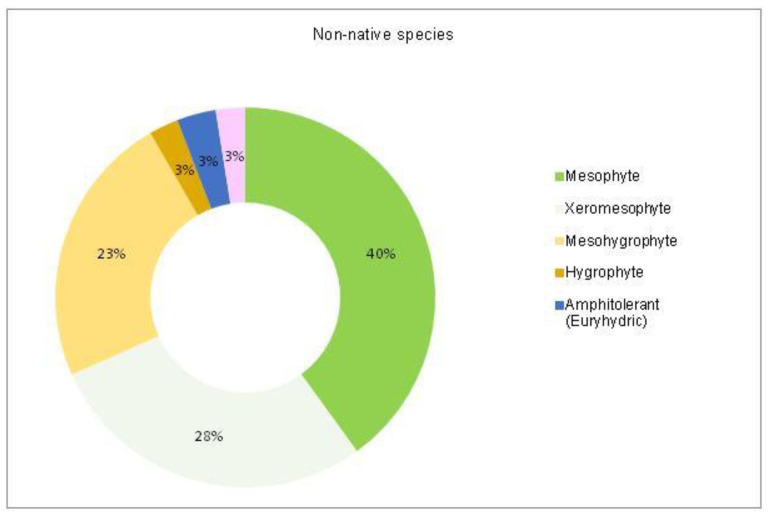
The spectrum of species requirements for moisture in the non-native dendroflora of the Botanic Park from Timișoara City.

**Figure 5:**
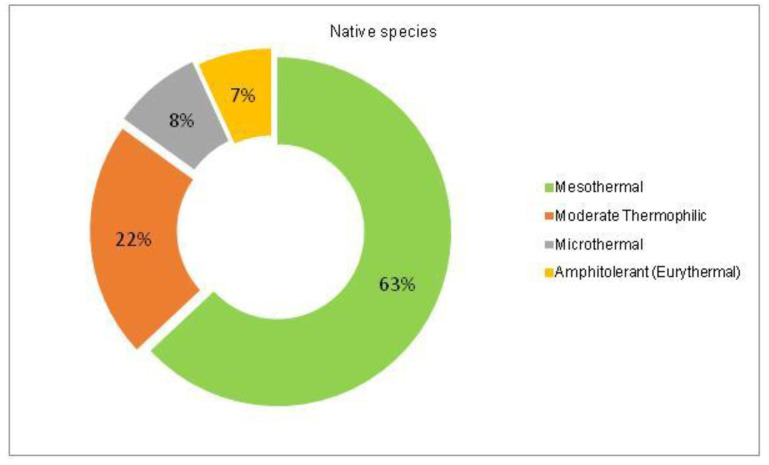
The spectrum of species requirements for temperature in the native dendroflora of the Botanic Park from Timișoara City.

**Figure 6:**
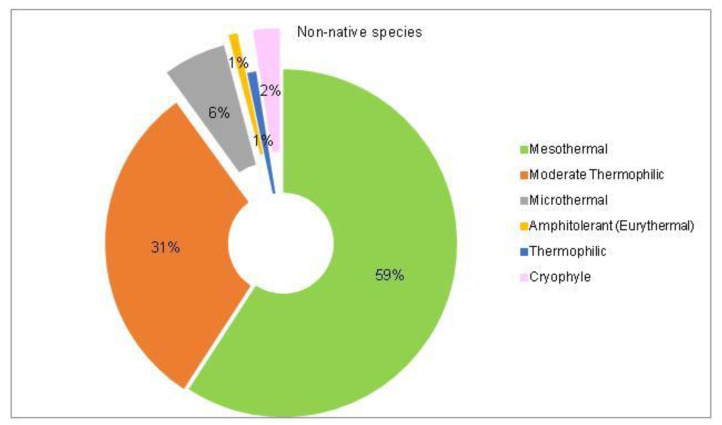
The spectrum of species requirements for temperature in the non-native dendroflora of the Botanic Park from Timișoara City.

**Figure 7:**
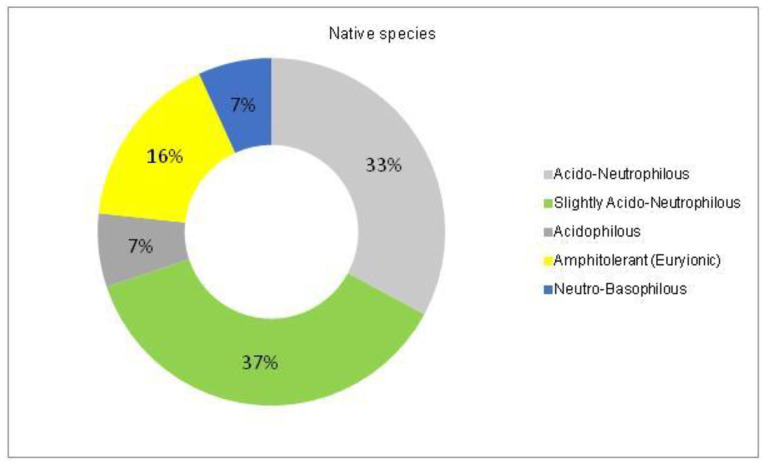
The spectrum of species requirements for soil pH in the native dendroflora of the Botanic Park from Timișoara City.

**Figure 8:**
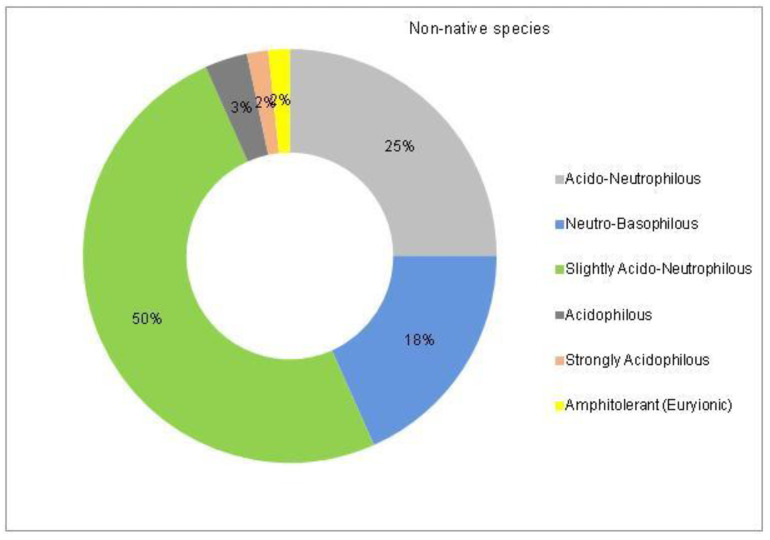
The spectrum of species requirements for soil pH in the non-native dendroflora of the Botanic Park from Timișoara City.

The analysis of the species requirements for the factors moisture, temperature and soil pH showed the dominance of mesophyte, mesothermal and slightly acido-neutrophilous species both in the native and non-native dendroflora (Figure 3, Figure 4, Figure 5, Figure 6, Figure 7 and Figure 8).

The dominance of the mesophytes in the analyzed urban park is compliant with the local temperate climate. The percentages of the mesohygrophytes differ majorly in the native dendroflora (9%) compared with the non-native one (23%) (Figure 3 and Figure 4).

The acclimation capacity of the mesohygrophytes in the studied arboretum is possible because it is sustained by the moisture regime offered by the park management. There was found in the natural forest ecosystems that changes of the moistening degree could induce the transformation of the plant cover structure, such as appearance of the hygrophytes or the increase of the mesohygrophytes role in the phytocoenosis [31]. Other studies showed the contribution of the environmental factors upon the vegetation shifting from one type of moisture requirement to another. A study regarding the riparian vegetation in China, indicated the vegetation shifting from xerophytes to mesophytes or even to hygrophytes in areas where the flooding fluctuations in a vegetal biocoenosis where the annual herbs were the dominant life form [32].

Within the native dendroflora, six plant species from the total 73 species, meaning 8%, are microthermal (Figure 5): 2 species are mesophanerophytes (*Salix viminalis* and *Spiraea salicifolia*), and 4 species are megaphanerophytes (*Alnus incana, Betula pendula, Pinus mugo*, and *Sorbus aucuparia)* (Table 1).

The acclimation capacity of the microthermal species in cities, which are known as heat islands, in contrast to their surrounding environments, has been previously reported and is possible in the cooler microhabitats provided by the urban matrix [33,34]: ventilation corridors [35], reflective building materials, shading-oriented design of the buildings or other elements of urban morphology, like building geometry or building height, suggesting that complex built context is more efficient in urban cooling due to its increased shading potential [36]. *Salix viminalis* is a microthermal species successfully adapted to the temperate climates and urban environment [37], which demonstrated real contributions for the urban environment, due to its potential to grow on metal-contaminated urban soil and for its potential of metal phytoextraction (Zn and Cd) from the contaminated urban soil and thus of phytoremediation of the urban soil [38,39]. *Spiraea salicifolia* is a microthermal species with high efficiency in carbon sequestration in the urban green spaces [40]. In the arctic urban climates, *Spiraea salicifolia* has shown low resistance to winter freezing, although it develops physiological adaptations to mitigate the winter hardiness by stocking sucrose in their tissues in autumn, to be later released during the winter period [41]. *Alnus incana* and *Sorbus aucuparia* are important species for the urban environment, these species have bioaccumulation potential of the cesium, transferring it from the urban topsoil in their biomass [42], so that the adaption of these microthermal tree species to the temperate climate of the analyzed site ends in an important ecosystem services. *Alnus incana* is also an efficient species in particulate matter removal [43] and phytoremediation of the organic hydrocarbons [44] in the urban areas. Other studies showed competition in the urban environments between *Alnus incana, Sorbus aucuparia* and *Betula pendula*: the decrease in abundance of the *Sorbus aucuparia* determined the abundance increase of *Alnus incana* and the decrease of *Betula pendula* in an urban forest from Finland [45]. *Alnus incana* has also other benefits for the urban soils, contributing to the nutrient cycles, through nitrogen fixation in the roots, via symbiotic *Frankia,* even in polluted soils [46]. *Betula pendula* is a species largely encountered in the natural areas of Northeastern and Central Europe and is a pioneer species in the natural ecological successions [47], with characteristics emerging from this quality: fast growing and thus lower mechanical properties, which make this species more vulnerable to the stressing micro-environmental, local conditions of the site, like winds [48]. From this point of view, it might be possible that *Betula pendula* be a more successful species in cities, in organized arboretums, where the winds are attenuated by the buildings, because some studies indicate that the local climatic conditions have more significant contribution to the mechanical stability of this species in cities than its mechanical properties, since no significant differences between the mechanical properties of this species in the urban areas versus in the forests has been found [47]. However, this mechanical sensitivity of the *Betula pendula* species is outweighed by its aesthetic and ecological benefits for the urban environment [49]. *Pinus mugo,* although a microthermal species, is well acclimated in cities due to its resistance to drought, and it is resistant in the arid and semi-arid regions. The mechanisms which mediate these adaptions, making the *Pinus mugo* appropriate to be planted in dry and drought environments are physiological and biochemical and refer to: shoot biomass, chlorophyll and carotenoid contents, water content, electrolyte leakage, sugar content, antioxidant enzymes, nutrient content, fatty acid content, protein content, osmosis [50]. The acclimative capacity of the above mentioned microthermal tree species to the conditions of the temperate climate is proven by their presence and continuity in the studied urban site as native species of the Romanian flora, and may represent a potential solution to certain urban environmental issues, and thus a source of ecosystem services for the studied urban environment.

The alien dendroflora of the studied urban park is characterized majorly through mesothermal and moderate termophilic species, as expected for a temperate climate site, but also through few microthermal—7 (6%) species and respectively through 3 (2%) cryophyle species (Figure 6). The microthermal species are: *Berberis julianae, Berberis stenophylla*, *Berberis thunbergii*, *Berberis haoi*, *Abies concolor*, *Pinus excela*, *Pinus wallichiana*, and the cryophyle tree species are: *Abies pinsapo*, *Picea pungens*, and *Tsuga canadensis*. These species has been previously reported in the temperate-climate urban parks. The acclimation of the plant species with low-temperature requirements in temperate zones can be possible because the tolerance to cold stress can be lost after exposure to warmer climate, through faster processes than the acquiring one [51]. This mechanism enables the plants species that possess it to have great responsiveness and adaptability to different types of climates and has been observed also in plants with Arctic origin [52].

Some studies have shown a decline of the native tree species across Europe [53], and the acclimation of the non-native tree species seems to be one of the reasons of their invasive potential threatening the native species. However, an analysis about the distribution of the woody invaders from contrasting climatic origins across the urban-rural gradient in oceanic Europe, showed that the woody alien plants with warmer native requirements are more present in the urban local climates [33].

A statistically significant association (Chi-square test) was found between the moisture and temperature requirements of species in both native (χ^2^ (15, *n* = 73) = 49.11, *p* < 0.001) and non-native dendroflora (χ^2^ (25, *n* = 120) = 65.91, *p* < 0.001) of the studied urban park. The response of the urban vegetation to the urban temperature oscillations (frequent and prolonged heat-waves) in the actual context of climate changes is a complex phenomenon involving structural and physiological mechanisms, not always in accordance to the native characteristics of the species. The woody vegetation reacts dynamically to keep its resilience to temperature variations, by changes in the stomatal conductance, leaf water potential, photosynthesis efficiency, respiration and evapotranspiration [54] or through physical and mechanical strategies able to modify the surrounding microclimate, like reciprocal leaf shading [55]. Other mechanisms are the geographic distributional shifting, but in the case of the exotic species acclimated in the urban parks, the niche breadth (referring to the range of ecological factors tolerated by the species) of their natural distribution is not respected, so that the physiological mechanisms remain the primary strategy adopted for coping with and adapting to the local environmental conditions.

Another statistically significant associations (Chi-square test) was found between the temperature preferences and soil pH preferences of the studied dendroflora, both native (χ^2^ (12, *n* = 73) = 40.55, *p* < 0.001) and non-native (χ^2^ (25, *n* = 120) = 59.25, *p* < 0.001). This correlation is important in assessing the potential of the identified species to develop adapting relationships to the urban environmental factors, because the temperature [56] and the soil pH are closely influencing the plant functional traits [57] in their acclimation and adaption strategies. However, the pH variability of the urban soils is high and therefore correlations between it and other plant features are difficult to be obtained. A study concerning several Mediterranean evergreen woods showed not statistically significant correlations of the species preferences for soil pH with other plant traits like temperature and precipitations [58]. Other studies carried in human-controlled ecosystems, such as arable lands, showed correlations between the plant preferences for soil pH and site elevation, and respectively between plant preferences for temperature and elevation or season [59,60].

In the studied dendrological park, the main part of the woody vegetation (37% of native dendroflora and 50% of non-native dendroflora) natively prefers the slightly acido-neutrophilous soils (Figure 7 and Figure 8), but this fact does not transform the respective flora into an indicative one of soil pH, because there are several studies which indicate that the native preference of the woody species for soil pH does not accurately describe the in situ value of soil pH [61,62] due to the extensive mechanisms of adaption to the environmental local factors exhibited by the species. For example, due to the irrigation practices imposed by the management of the urban parks [63] or because of other factors like the deicing salts which are used in winter in the temperate zones to defrost the urban streets and which are indicated as the main cause of salt stress in the urban environments [64], the urban soil faces with salt accumulation, an environmental soil factor to which the urban plants must be adapted during their acclimation or to exceed the limits imposed by their native requirements.

The pH variability of the urban soils is a phenomenon widely appearing in the urban areas and this has been described in many urban sites [65]. Because of this limitation, correlations between the native preferences of the plants regarding the environmental factors become viable options to be considered, especially in the ecological studies regarding the acclimation and adaption of the plant species in the urban area, where the soil pH reaches high variability. For example, the plant preferences for pH have been proven to be a good predictor of species richness [66].

In the studied urban arboretum, within the acclimation process of the non-native dendroflora, 37% of species exceeded their native requirements for moisture, 41% for temperature, and 50% for soil pH.

**Figure 9:**
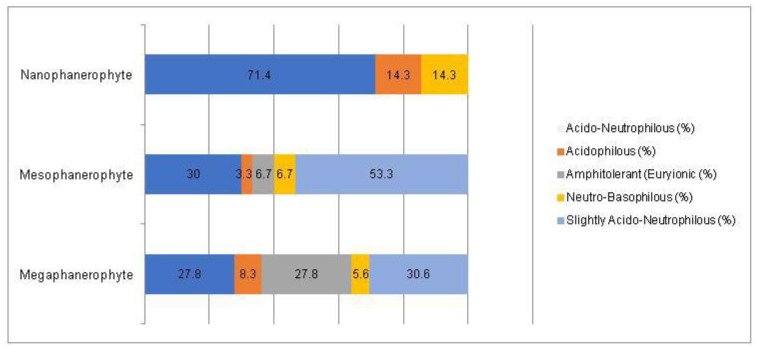
Contingence diagram regarding the significant association (Chi-square test, χ^2^ (8, *n* = 73) = 16.27, *p* = 0.039) and distribution of the soil pH-requirement among the plant life-form spectrum in the native woody species.

**Figure 10:**
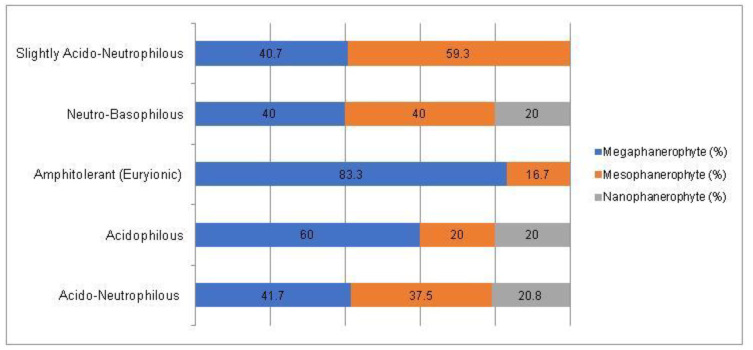
Contingence diagram regarding the significant association (Chi-square test, χ^2^ (8, *n* = 73) = 16.27, *p* = 0.039) and distribution of the plant life-forms among the soil pH-requirement spectrum in the native woody species.

**Figure 11:**
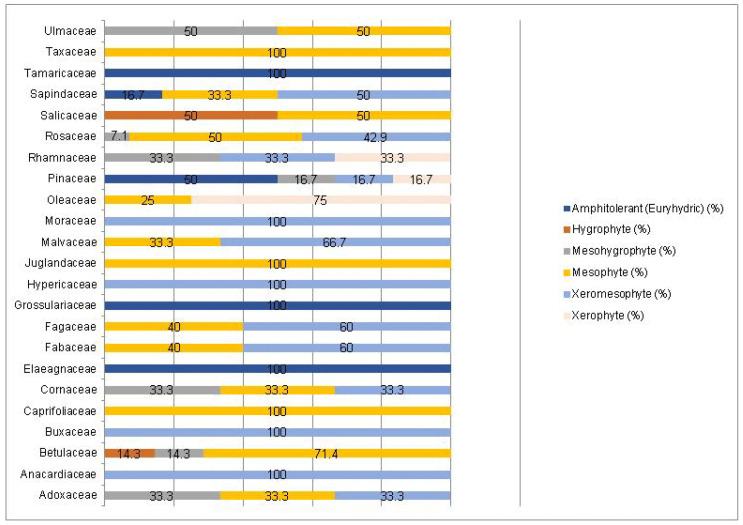
Contingence diagram regarding the significant association (Chi-square test, χ^2^ (110, *n* = 73) = 139.72, *p* = 0.029) and distribution of the plant moisture-requirements among the plant-families spectrum in the native woody species.

**Figure 12:**
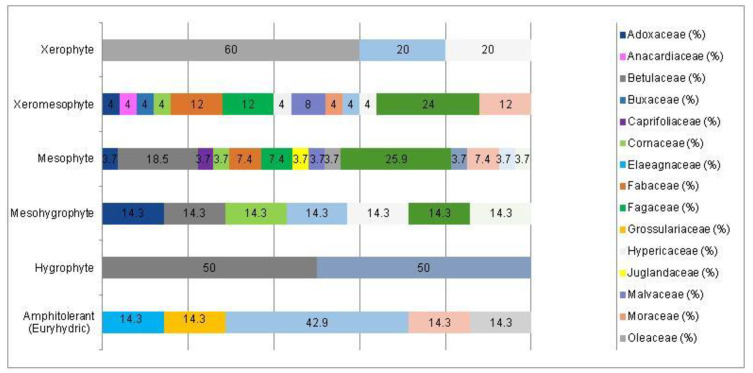
Contingence diagram regarding the significant association (Chi-square test, χ^2^ (110, *n* = 73) = 139.72, *p* = 0.029) and distribution of the plant-families among the plant moisture-requirement spectrum in the native woody species.

A Chi-square test has been conducted to determine whether there is an association between the studied factors in the native dendroflora of the studied urban green space. There was found significant associations between the plant life-form (megaphanerophyte, mesophanerophyte, nanophanerophyte) and the ecological plant requirements for soil pH (χ^2^ (8, *n* = 73) = 16.27, *p* = 0.039) (Figure 9 and Figure 10), and respectively between the plant family and the plant requirement for moisture (χ^2^ (110, *n* = 73) = 139.72, *p* = 0.029) (Figure 11 and Figure 12).

**Figure 13:**
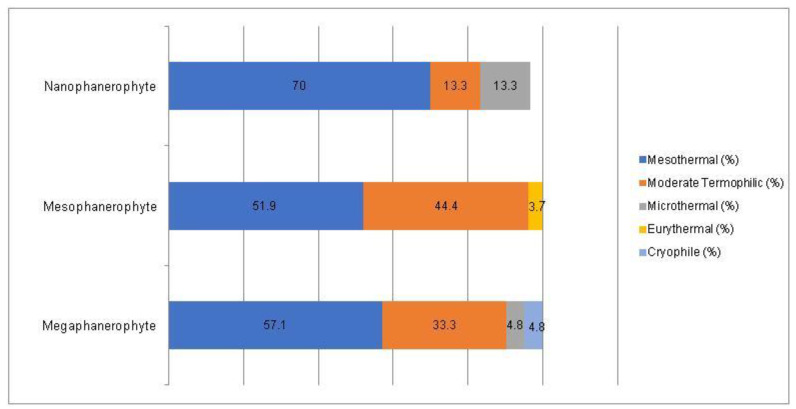
Contingence diagram regarding the significant association (Chi-square test, χ^2^ (10, *n* = 120) = 19.36, *p* = 0.036) and distribution of the plant temperature-requirement among the plant life-form spectrum in the non-native woody species.

**Figure 14:**
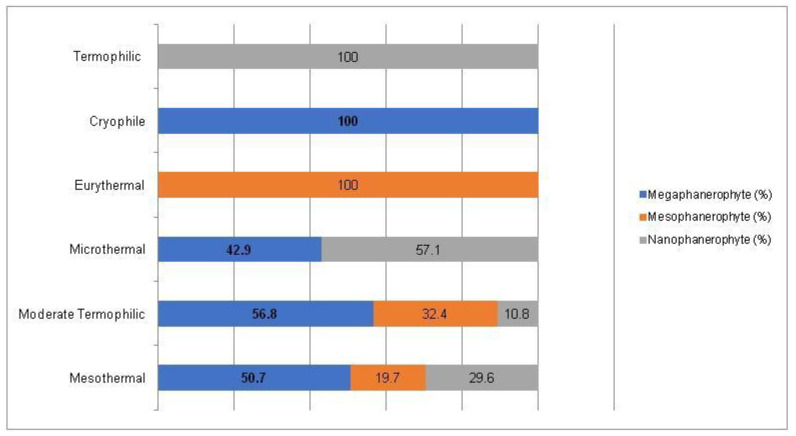
Contingence diagram regarding the significant association (Chi-square test, χ^2^ (10, *n* = 120) = 19.36, *p* = 0.036) and distribution of the life-forms among the plant temperature-requirement spectrum in the non-native woody species.

For the non-native woody species, the Chi-square test has shown a statistically significant association between the plant life-form and the temperature preferences for all three types of plant life-forms (megaphanerophytes, mesophanerophytes and nanophanerophytes) present in the studied arboretum (Chi-square test, χ^2^ (10, *n* = 120) = 19.36, *p* = 0.036) (Figure 13 and Figure 14).

The fact that plant life-form is associated with the plant pH requirement in the native dendroflora, while in the alien dendroflora is associated with the temperature requirement results from the evolutionary adaption strategies of the species during their historical survival in various environments. The introduced species face often shifting ecological requirements necessitating fast acclimation adaption, and the temperature is a first filter in species selection and spread, unlike native species which evolves in situ. Because of their long life span, the woody species tend to specialize more in their native soil pH because this is a factor involved in multiple ecological interactions and a driver of the ecological niches. Thus, the evolutionary pressure shapes the woody species to match the soil conditions of their habitats, ensuring the species long time resilience.

## 3. Materials and Methods

### 3.1. Research Site

**Figure 15:**
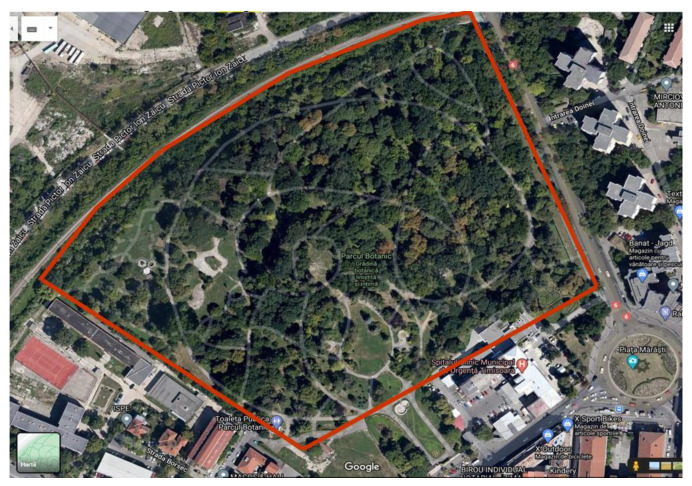
The Botanic Park of Timişoara (45°45′18″ N, 21°13′28″ E) delimited within the red borders (Google Maps capture).

The research was conducted in the Botanic Park of Timișoara, Timiș County, Romania (45°45′18″ N, 21°13′28″ E) (Figure 15), located in the north of the Bega River, which flows through the city. The Botanic Park of Timișoara has been established between years 1986–1990, by the Romanian architect Silvia Grumeza, under the name Botanical Garden of Timișoara, and it was opened to visitors on 29 June 1986 [67]. From the beginning, it was designed as an arboretum (dendrological park). Initially, over 1650 plant species with diverse origins were planted here [67]. The park covers an area of 8.41 hectares [68]. Since 1986, when the Botanic Park of Timișoara has been established, the human intervention at soil level is mainly for the care of the few herbaceous decorative plants, therefore nowadays the soil park is considered a semi-natural soil. Since 1995, by County Council Decision no. 19/23 February 1995, the Botanic Park was declared a protected natural area—for the conservation of biodiversity, the gene pool, the ecological reserve, and to maintain the ecological balance in Timiș County, with Timișoara City Hall designated as its manager. The Botanic Park of Timișoara is a protected area with two main objectives: (1) the conservation and development of the dendrological collection and (2) the conservation and enhancement of landscapes, with the possibility of being visited for scientific, touristic, educational, and recreational-social purposes [67].

### 3.2. Research Methodology

The inventory of the woody plant species present in the Botanic Park of Timișoara have been provided upon request by the Timișoara City Hall—Office of Recreational Green Spaces, the authority who sourced the work “Local Register of Green Spaces—Timișoara Municipality” [69]. Totally, the dendrofloristic list comprised 193 species (73 species are native to Romania and 120 species are alien cultural species). All species have been described using the working methodology focused on three goals:

1. Establishing the categories of plant life-forms (bioforms, biological forms) specific to the studied dendroflora according to C. Raunkiaer’s classification [70]. The plant life-forms are the expression of the convergent evolution of different species, which gives them similar morphological, structural, and physiological characteristics [71]. The method has been chosen for the purpose of the present research because the delineation of bioforms in plant ecology is based on grouping species by their survival strategies during periods with critical ecological factors, regardless of their taxonomic affiliation. The most widely accepted classification is that of C. Raunkiaer [1934], which is primarily based on how plant’s regenerative structures are protected during the unfavorable season, specifically the position of the renewal organs (buds). Thus, in the delineation of plant life forms (bioforms), the key factor is the level (relative to the soil surface) at which the tissues that ensure the plant’s perennity are found.

2. Classification of plant species into categories of phytogeographical elements (geoelements, chorology) according to a methodology provided by several Romanian authorities in the field [72,73,74,75]. The geoelements serve to designate categories of plant species that are more or less distantly related phylogenetically, which, during the process of speciation, have occupied the same geographical region, and then followed specific migration paths and coenotic integration towards the formation of their current distribution ranges [72,73,74,75].

3. Identifying the ecological requirements of the studied plant species for factors such as moisture, temperature, and soil pH according to the methodology proposed by Sanda et al. [1983]. This methodology is in the sense of Ellenberg scale of plant preferences for the same ecological factors [76] and consists of attributing an ecological preference index and respectively an ecological significance description for each species depending on its preferences for moisture, temperature, and soil pH (Table 3).

For the spontaneous dendroflora there was analyzed the chorological spectrum which shows the geographic distribution of the plant species, but this characteristic has been dropped for the non-native dendroflora because of the great heterogeneity of this group and the difficulty (limited accessible resources) in establishing the phytogeographic element for each non-native species with accuracy, which could affect the reliability of the study. Instead this, there was chosen to be analyzed the acclimation of the non-native species to the local environmental conditions only by studying other characteristics of them such as the habitat requirements for moisture, temperature and soil pH, to conclude about the adabtability of the non-native species to the host urban ecosystem.

The statistical processing and interpretations of data have been performed using the IBM Software SPSS version 28.0., and the graphical representations have been outputted using the Microsoft Office Excel (2007) tool.

## 4. Conclusions

The species composition of the studied urban arboretum indicates high species richness. The species are taxonomically and biogeographically diverse, both native and non-native, suggesting the great ability of the identified woody species in acclimating to the temperate environment characteristic to the studied site.

The dominant dendroflora is mesophyte, mesothermal and slightly acido-neutrophilous. The evolutionary and acclimating pressure shaped the woody species of the urban park to match the habitat conditions according to their native requirements for moisture, temperature and soil pH, or shifting them. Therefore, in the acclimation process of the non-native dendroflora, 37% of species exceeded their native requirements for moisture, 41% for temperature, and 50% for soil pH. With the same purpose, there was found that in the acclimation process, the plant life-form is relevant both in the native and alien dendroflora, and the plant family is relevant only in the native dendroflora. These findings sustain the sustainability of the studied urban park and its long time resilience, ensuring its potential in providing local ecosystem services.

## Figures and Tables

**Table 1:** Ecological traits of the native dendroflora of the Botanic Park of Timișoara City.

No.	Species	Family	Monophyletic Group *	Plant Chorology (Phytogeographic Elements)	Plant Life-Forms	Moisture Requirement	Temperature Requirement	Soil pH Requirement
1	*Sambucus nigra*	Adoxaceae	A	European	Mesophanerophyte	Mesophyte	Mesothermal	Acido-Neutrophilous
2	*Viburnum lantana*	Adoxaceae	A	Mediterranean—Central European	Mesophanerophyte	Xeromesophyte	Mesothermal	Slightly Acido-Neutrophilous
3	*Viburnum opulus*	Adoxaceae	A	Circumpolar	Mesophanerophyte	Mesohygrophyte	Mesothermal	Slightly Acido-Neutrophilous
4	*Cotinus coggygria*	Anacardiaceae	A	Mediterranean	Mesophanerophyte	Xeromesophyte	Moderate Thermophilic	Slightly Acido-Neutrophilous
5	*Alnus glutinosa*	Betulaceae	A	Eurasian	Megaphanerophyte	Hygrophyte	Mesothermal	Acido-Neutrophilous
6	*Alnus incana*	Betulaceae	A	Eurasian	Megaphanerophyte	Mesohygrophyte	Microthermal	Slightly Acido-Neutrophilous
7	*Betula pendula*	Betulaceae	A	Eurasian	Megaphanerophyte	Mesophyte	Microthermal	Acidophilous
8	*Carpinus betulus*	Betulaceae	A	European	Megaphanerophyte	Mesophyte	Mesothermal	Acido-Neutrophilous
9	*Corylus avellana*	Betulaceae	A	European	Mesophanerophyte	Mesophyte	Mesothermal	Acido-Neutrophilous
10	*Corylus colurna*	Betulaceae	A	Eurasian	Megaphanerophyte	Mesophyte	Moderate Thermophilic	Slightly Acido-Neutrophilous
11	*Fagus sylvatica*	Betulaceae	A	European	Megaphanerophyte	Mesophyte	Mesothermal	Amphitolerant (Euryionic)
12	*Buxus sempervirens*	Buxaceae	A	Eurasian	Nanophanerophyte	Xeromesophyte	Mesothermal	Acido-Neutrophilous
13	*Lonicera xylosteum*	Caprifoliaceae	A	Eurasian	Mesophanerophyte	Mesophyte	Mesothermal	Slightly Acido-Neutrophilous
14	*Cornus alba*	Cornaceae	A	Eurasian	Nanophanerophyte	Mesohygrophyte	Moderate Thermophilic	Acido-Neutrophilous
15	*Cornus mas*	Cornaceae	A	Pontic- Mediterranean—Central European	Mesophanerophyte	Xeromesophyte	Mesothermal	Slightly Acido-Neutrophilous
16	*Cornus sanguinea*	Cornaceae	A	Central European	Mesophanerophyte	Mesophyte	Mesothermal	Slightly Acido-Neutrophilous
17	*Hippophae rhamnoides*	Elaeagnaceae	A	Eurasian	Mesophanerophyte	Amphitolerant (Euryhydric)	Mesothermal	Slightly Acido-Neutrophilous
18	*Amorpha fruticosa*	Fabaceae	A	Adventive	Mesophanerophyte	Mesophyte	Moderate Thermophilic	Amphitolerant (Euryionic)
19	*Cercis siliquastrum*	Fabaceae	A	Eurasian	Mesophanerophyte	Xeromesophyte	Moderate Thermophilic	Acido-Neutrophilous
20	*Laburnum anagyroides*	Fabaceae	A	Balkan	Mesophanerophyte	Mesophyte	Mesothermal	Acido-Neutrophilous
21	*Robinia pseudoacacia*	Fabaceae	A	Adventive	Megaphanerophyte	Xeromesophyte	Moderate Thermophilic	Amphitolerant (Euryionic)
22	*Sarothamnus scoparius*	Fabaceae	A	Atlantic- Mediterranean—Central European	Nanophanerophyte	Xeromesophyte	Mesothermal	Acidophilous
23	*Castanea sativa*	Fagaceae	A	Mediterranean	Megaphanerophyte	Xeromesophyte	Moderate Thermophilic	Acidophilous
24	*Quercus cerris*	Fagaceae	A	Mediterranean	Megaphanerophyte	Xeromesophyte	Mesothermal	Acido-Neutrophilous
25	*Quercus macranthera*	Fagaceae	A	European- Anatolian- Caucasian	Megaphanerophyte	Mesophyte	Moderate Thermophilic	Neutro-Basophilous
26	*Quercus petraea*	Fagaceae	A	European	Megaphanerophyte	Xeromesophyte	Mesothermal	Amphitolerant (Euryionic)
27	*Quercus robur*	Fagaceae	A	European	Megaphanerophyte	Mesophyte	Mesothermal	Amphitolerant (Euryionic)
28	*Ribes nigrum*	Grossulariaceae	A	Eurasian	Mesophanerophyte	Amphitolerant (Euryhydric)	Amphitolerant (Eurythermal)	Acido-Neutrophilous
29	*Hypericum androsaemum*	Hypericaceae	A	Eurasian	Nanophanerophyte	Xeromesophyte	Moderate Thermophilic	Acido-Neutrophilous
30	*Juglans regia*	Juglandaceae	A	Carpathian- Balkan- Anatolian- Caucasian	Megaphanerophyte	Mesophyte	Moderate Thermophilic	Slightly Acido-Neutrophilous
31	*Tilia cordata*	Malvaceae	A	European	Megaphanerophyte	Mesophyte	Mesothermal	Acido-Neutrophilous
32	*Tilia platyphyllos*	Malvaceae	A	Central European	Megaphanerophyte	Xeromesophyte	Mesothermal	Slightly Acido-Neutrophilous
33	*Tilia tomentosa*	Malvaceae	A	Balkan	Megaphanerophyte	Xeromesophyte	Mesothermal	Acido-Neutrophilous
34	*Morus alba*	Moraceae	A	Adventive	Megaphanerophyte	Xeromesophyte	Mesothermal	Slightly Acido-Neutrophilous
35	*Fraxinus excelsior*	Oleaceae	A	European	Megaphanerophyte	Mesophyte	Mesothermal	Slightly Acido-Neutrophilous
36	*Fraxinus ornus*	Oleaceae	A	Mediterranean	Mesophanerophyte	Xerophyte	Mesothermal	Neutro-Basophilous
37	*Jasminum fruticans*	Oleaceae	A	Mediterranean	Mesophanerophyte	Xerophyte	Moderate Thermophilic	Slightly Acido-Neutrophilous
38	*Syringa vulgaris*	Oleaceae	A	Balkan- Anatolian	Mesophanerophyte	Xerophyte	Moderate Thermophilic	Slightly Acido-Neutrophilous
39	*Abies alba*	Pinaceae	A	Central European	Megaphanerophyte	Mesohygrophyte	Mesothermal	Amphitolerant (Euryionic)
40	*Larix decidua*	Pinaceae	A	Central European- Carpathian-Sudetic	Megaphanerophyte	Xeromesophyte	Amphitolerant (Eurythermal)	Amphitolerant (Euryionic)
41	*Picea abies*	Pinaceae	A	European	Megaphanerophyte	Amphitolerant (Euryhydric)	Amphitolerant (Eurythermal)	Amphitolerant (Euryionic)
42	*Pinus mugo*	Pinaceae	A	European	Megaphanerophyte	Amphitolerant (Euryhydric)	Microthermal	Amphitolerant (Euryionic)
43	*Pinus nigra*	Pinaceae	A	Carpathian	Megaphanerophyte	Xerophyte	Moderate Thermophilic	Slightly Acido-Neutrophilous
44	*Pinus sylvestris*	Pinaceae	G	Eurasian	Megaphanerophyte	Amphitolerant (Euryhydric)	Amphitolerant (Eurythermal)	Amphitolerant (Euryionic)
45	*Rhamnus cathartica*	Rhamnaceae	A	Eurasian	Mesophanerophyte	Xeromesophyte	Mesothermal	Slightly Acido-Neutrophilous
46	*Frangula rupestris*	Rhamnaceae	A	European	Nanophanerophyte	Mesohygrophyte	Mesothermal	Acido-Neutrophilous
47	*Ziziphus jujuba*	Rhamnaceae	A	Mediterranean	Mesophanerophyte	Xerophyte	Moderate Thermophilic	Neutro-Basophilous
48	*Cotoneaster integerrimus*	Rosaceae	A	Eurasian	Nanophanerophyte	Xeromesophyte	Mesothermal	Neutro-Basophilous
49	*Crataegus laevigata*	Rosaceae	A	Central European	Mesophanerophyte	Mesophyte	Mesothermal	Acido-Neutrophilous
50	*Crataegus monogyna*	Rosaceae	A	European	Mesophanerophyte	Xeromesophyte	Mesothermal	Acido-Neutrophilous
51	*Crataegus pentagyn*.	Rosaceae	A	Mediterranean	Mesophanerophyte	Mesophyte	Mesothermal	Acido-Neutrophilous
52	*Malus sylvestris*	Rosaceae	A	European	Mesophanerophyte	Mesophyte	Mesothermal	Slightly Acido-Neutrophilous
53	*Prunus avium*	Rosaceae	A	European	Mesophanerophyte	Mesophyte	Mesothermal	Acido-Neutrophilous
54	*Prunus cerasifera*	Rosaceae	A	Eurasian	Mesophanerophyte	Xeromesophyte	Moderate Thermophilic	Amphitolerant (Euryionic)
55	*Prunus padus*	Rosaceae	A	Eurasian	Megaphanerophyte	Mesophyte	Mesothermal	Slightly Acido-Neutrophilous
56	*Pyrus pyraster*	Rosaceae	A	European	Mesophanerophyte	Xeromesophyte	Mesothermal	Slightly Acido-Neutrophilous
57	*Rosa canina*	Rosaceae	A	European	Nanophanerophyte	Xeromesophyte	Mesothermal	Acido-Neutrophilous
58	*Sorbus aria*	Rosaceae	A	European	Megaphanerophyte	Mesophyte	Mesothermal	Neutro-Basophilous
59	*Sorbus aucuparia*	Rosaceae	A	European	Megaphanerophyte	Mesophyte	Microthermal	Acidophilous
60	*Sorbus torminalis*	Rosaceae	A	European	Megaphanerophyte	Xeromesophyte	Mesothermal	Slightly Acido-Neutrophilous
61	*Spiraea salicifolia*	Rosaceae	A	Eurasian	Mesophanerophyte	Mesohygrophyte	Microthermal	Acidophilous
62	*Salix viminalis*	Salicaceae	A	Eurasian	Mesophanerophyte	Hygrophyte	Microthermal	Slightly Acido-Neutrophilous
63	*Populus alba*	Salicaceae	A	Eurasian	Megaphanerophyte	Mesophyte	Mesothermal	Acido-Neutrophilous
64	*Acer campestre*	Sapindaceae	A	European	Megaphanerophyte	Xeromesophyte	Mesothermal	Acido-Neutrophilous
65	*Acer monspessulanum*	Sapindaceae	A	Mediterranean	Megaphanerophyte	Xeromesophyte	Moderate Thermophilic	Slightly Acido-Neutrophilous
66	*Acer platanoides*	Sapindaceae	A	Eurasian	Megaphanerophyte	Mesophyte	Mesothermal	Acido-Neutrophilous
67	*Acer pseudoplatanus*	Sapindaceae	A	Central European	Megaphanerophyte	Mesophyte	Mesothermal	Acido-Neutrophilous
68	*Acer tataricum*	Sapindaceae	A	European	Mesophanerophyte	Xeromesophyte	Mesothermal	Slightly Acido-Neutrophilous
69	*Ailanthus altissima*	Sapindaceae	A	Adventive	Megaphanerophyte	Amphitolerant (Euryhydric)	Amphitolerant (Eurythermal)	Amphitolerant (Euryionic)
70	*Tamarix ramosissima*	Tamaricaceae	A	Eurasian	Mesophanerophyte	Amphitolerant (Euryhydric)	Mesothermal	Slightly Acido-Neutrophilous
71	*Taxus baccata*	Taxaceae	G	European	Mesophanerophyte	Mesophyte	Mesothermal	Slightly Acido-Neutrophilous
72	*Ulmus glabra*	Ulmaceae	A	Eurasian	Megaphanerophyte	Mesohygrophyte	Mesothermal	Acido-Neutrophilous
73	*Ulmus minor*	Ulmaceae	A	Eurasian	Megaphanerophyte	Mesophyte	Mesothermal	Slightly Acido-Neutrophilous

* A = Angiosperm; G = Gymnosperm.

**Table 2:** Ecological traits of the non-native dendroflora of the Botanic Park of Timișoara City.

No.	Species	Family	Monophyletic Group *	Plant Life-Forms	Moisture Requirement	Temperature Requirement	Soil pH Requirement
1	*Liquidambar styraciflua*	Altingiaceae	A	Megaphanerophyte	Mesophyte	Mesothermal	Acido-Neutrophilous
2	*Rhus semialata*	Anacardiaceae	A	Mesophanerophyte	Mesophyte	Mesothermal	Slightly Acido-Neutrophilous
3	*Rhus typhina*	Anacardiaceae	A	Mesophanerophyte	Xeromesophyte	Amphitolerant (Eurythermal)	Acido-Neutrophilous
4	*Kalopanax septemlobus*	Araliaceae	A	Megaphanerophyte	Mesophyte	Moderate Thermophilic	Acido-Neutrophilous
5	*Berberis julianae*	Berberidaceae	A	Nanophanerophyte	Xeromesophyte	Microthermal	Strongly Acidophilous
6	*Berberis stenophylla*	Berberidaceae	A	Nanophanerophyte	Xeromesophyte	Microthermal	Strongly Acidophilous
7	*Berberis thunbergii*	Berberidaceae	A	Nanophanerophyte	Xeromesophyte	Microthermal	Amphitolerant (Euryionic)
8	*Berberis haoi*	Berberidaceae	A	Nanophanerophyte	Xeromesophyte	Microthermal	Acidophilous
9	*Mahonia aquifolium*	Berberidaceae	A	Nanophanerophyte	Mesophyte	Mesothermal	Amphitolerant (Euryionic)
10	*Catalpa bignonioides*	Bignoniaceae	A	Megaphanerophyte	Mesophyte	Mesothermal	Acido-Neutrophilous
11	*Catalpa ovata*	Bignoniaceae	A	Megaphanerophyte	Mesophyte	Mesothermal	Slightly Acido-Neutrophilous
12	*Kolkwitzia amabilis*	Caprifoliaceae	A	Mesophanerophyte	Mesophyte	Mesothermal	Acido-Neutrophilous
13	*Lonicera fragrantissima*	Caprifoliaceae	A	Nanophanerophyte	Mesophyte	Mesothermal	Acido-Neutrophilous
14	*Lonicera tatarica*	Caprifoliaceae	A	Nanophanerophyte	Mesophyte	Mesothermal	Acidophilous
15	*Symphoricarpos albus*	Caprifoliaceae	A	Nanophanerophyte	Mesohygrophyte	Mesothermal	Acido-Neutrophilous
16	*Weigela florida*	Caprifoliaceae	A	Nanophanerophyte	Mesophyte	Mesothermal	Slightly Acido-Neutrophilous
17	*Euonymus bungeanus*	Celastraceae	A	Nanophanerophyte	Mesophyte	Mesothermal	Neutro-Basophilous
18	*Cercidiphyllum japonicum*	Cercidiphyllaceae	A	Megaphanerophyte	Mesohygrophyte	Moderate Thermophilic	Acido-Neutrophilous
19	*Chamaecyparis lawsoniana*	Cupressaceae	G	Megaphanerophyte	Mesohygrophyte	Mesothermal	Slightly Acido-Neutrophilous
20	*Chamaecyparis pisifera*	Cupressaceae	G	Megaphanerophyte	Mesohygrophyte	Mesothermal	Slightly Acido-Neutrophilous
21	*Cryptomeria japonica*	Cupressaceae	G	Megaphanerophyte	Mesohygrophyte	Moderate Thermophilic	Slightly Acido-Neutrophilous
22	*Cupressus arizonica*	Cupressaceae	G	Megaphanerophyte	Xeromesophyte	Moderate Thermophilic	Slightly Acido-Neutrophilous
23	*Juniperus chinensis*	Cupressaceae	G	Mesophanerophyte	Xeromesophyte	Mesothermal	Slightly Acido-Neutrophilous
24	*Juniperus horizontalis*	Cupressaceae	G	Nanophanerophyte	Xeromesophyte	Mesothermal	Slightly Acido-Neutrophilous
25	*Juniperus virginiana*	Cupressaceae	G	Mesophanerophyte	Xeromesophyte	Mesothermal	Slightly Acido-Neutrophilous
26	*Thuja occidentalis*	Cupressaceae	G	Megaphanerophyte	Mesophyte	Mesothermal	Slightly Acido-Neutrophilous
27	*Thuja occidentalis* var. *fastigiata*	Cupressaceae	G	Megaphanerophyte	Mesophyte	Mesothermal	Slightly Acido-Neutrophilous
28	*Thuja orientalis*	Cupressaceae	G	Megaphanerophyte	Mesophyte	Mesothermal	Slightly Acido-Neutrophilous
29	*Thuja plicata*	Cupressaceae	G	Megaphanerophyte	Mesophyte	Mesothermal	Acido-Neutrophilous
30	*Diospyros lotus*	Ebenaceae	A	Megaphanerophyte	Mesohygrophyte	Moderate Thermophilic	Slightly Acido-Neutrophilous
31	*Albizia julibrissin*	Fabaceae	A	Megaphanerophyte	Xeromesophyte	Moderate Thermophilic	Amphitolerant (Euryionic)
32	*Caragana arborescens*	Fabaceae	A	Megaphanerophyte	Xeromesophyte	Mesothermal	Slightly Acido-Neutrophilous
33	*Cercis chinensis*	Fabaceae	A	Megaphanerophyte	Mesophyte	Mesothermal	Acidophilous
34	*Gleditsia triacanthos*	Fabaceae	A	Megaphanerophyte	Xeromesophyte	Mesothermal	Neutro-Basophilous
35	*Gleditsia triacanthos* var. inermis	Fabaceae	A	Megaphanerophyte	Xeromesophyte	Mesothermal	Neutro-Basophilous
36	*Gymnocladus dioicus*	Fabaceae	A	Megaphanerophyte	Mesophyte	Mesothermal	Neutro-Basophilous
37	*Robinia hispida*	Fabaceae	A	Megaphanerophyte	Mesophyte	Mesothermal	Neutro-Basophilous
38	*Sophora japonica*	Fabaceae	A	Megaphanerophyte	Mesophyte	Moderate Thermophilic	Neutro-Basophilous
39	*Quercus rubra*	Fagaceae	A	Megaphanerophyte	Mesophyte	Mesothermal	Acido-Neutrophilous
40	*Quercus macrocarpa*	Fagaceae	A	Megaphanerophyte	Mesohygrophyte	Mesothermal	Neutro-Basophilous
41	*Ginkgo biloba*	Ginkgoaceae	G	Megaphanerophyte	Mesophyte	Mesothermal	Slightly Acido-Neutrophilous
42	*Deutzia scabra*	Hydrangeaceae	A	Mesophanerophyte	Mesohygrophyte	Mesothermal	Slightly Acido-Neutrophilous
43	*Philadelphus coronarius*	Hydrangeaceae	A	Mesophanerophyte	Mesohygrophyte	Mesothermal	Slightly Acido-Neutrophilous
44	*Philadelphus wilsonii*	Hydrangeaceae	A	Mesophanerophyte	Mesohygrophyte	Mesothermal	Slightly Acido-Neutrophilous
45	*Hypericum patulum*	Hypericaceae	A	Mesophanerophyte	Mesohygrophyte	Mesothermal	Slightly Acido-Neutrophilous
46	*Carya ovata*	Juglandaceae	A	Megaphanerophyte	Mesophyte	Moderate Thermophilic	Slightly Acido-Neutrophilous
47	*Juglans nigra*	Juglandaceae	A	Megaphanerophyte	Mesohygrophyte	Moderate Thermophilic	Slightly Acido-Neutrophilous
48	*Pterocarya fraxinifolia*	Juglandaceae	A	Nanophanerophyte	Hygrophyte	Mesothermal	Slightly Acido-Neutrophilous
49	*Punica granatum*	Lythraceae	A	Nanophanerophyte	Xeromesophyte	Moderate Thermophilic	Slightly Acido-Neutrophilous
50	*Liriodendron tulipifera*	Magnoliaceae	A	Megaphanerophyte	Mesohygrophyte	Moderate Thermophilic	Slightly Acido-Neutrophilous
51	*Magnolia kobus*	Magnoliaceae	A	Megaphanerophyte	Mesohygrophyte	Mesothermal	Acido-Neutrophilous
52	*Hibiscus syriacus*	Malvaceae	A	Nanophanerophyte	Mesophyte	Moderate Thermophilic	Slightly Acido-Neutrophilous
53	*Broussonetia papyrifera*	Moraceae	A	Megaphanerophyte	Xeromesophyte	Moderate Thermophilic	Slightly Acido-Neutrophilous
54	*Ficus carica*	Moraceae	A	Megaphanerophyte	Xeromesophyte	Moderate Thermophilic	Slightly Acido-Neutrophilous
55	*Maclura pomifera*	Moraceae	A	Megaphanerophyte	Xeromesophyte	Moderate Thermophilic	Slightly Acido-Neutrophilous
56	*Morus nigra*	Moraceae	A	Megaphanerophyte	Mesophyte	Moderate Thermophilic	Neutro-Basophilous
57	*Chionanthus retusus*	Oleaceae	A	Megaphanerophyte	Mesohygrophyte	Moderate Thermophilic	Slightly Acido-Neutrophilous
58	*Forsythia × intermedia*	Oleaceae	A	Nanophanerophyte	Mesophyte	Mesothermal	Neutro-Basophilous
59	*Fraxinus americana*	Oleaceae	A	Megaphanerophyte	Mesophyte	Mesothermal	Neutro-Basophilous
60	*Ligustrum ovalifolium*	Oleaceae	A	Megaphanerophyte	Mesophyte	Mesothermal	Slightly Acido-Neutrophilous
61	*Paeonia suffruticosa*	Paeoniaceae	A	Megaphanerophyte	Mesophyte	Mesothermal	Slightly Acido-Neutrophilous
62	*Paulownia tomentosa*	Paulowniaceae	A	Megaphanerophyte	Xeromesophyte	Moderate Thermophilic	Neutro-Basophilous
63	*Abies concolor*	Pinaceae	G	Megaphanerophyte	Mesohygrophyte	Microthermal	Slightly Acido-Neutrophilous
64	*Abies pinsapo*	Pinaceae	A	Megaphanerophyte	Mesohygrophyte	Cryophile	Acido-Neutrophilous
65	*Picea pungens*	Pinaceae	A	Megaphanerophyte	Mesohygrophyte	Cryophile	Acido-Neutrophilous
66	*Pinus excelsa*	Pinaceae	A	Megaphanerophyte	Xeromesophyte	Microthermal	Slightly Acido-Neutrophilous
67	*Pinus strobus*	Pinaceae	A	Megaphanerophyte	Mesophyte	Mesothermal	Acido-Neutrophilous
68	*Pinus wallichiana*	Pinaceae	A	Megaphanerophyte	Xeromesophyte	Microthermal	Slightly Acido-Neutrophilous
69	*Pseudotsuga menziesii*	Pinaceae	A	Megaphanerophyte	Mesohygrophyte	Mesothermal	Acido-Neutrophilous
70	*Pseudotsuga menziesii* var. *glauca*	Pinaceae	A	Megaphanerophyte	Mesohygrophyte	Mesothermal	Acido-Neutrophilous
71	*Tsuga canadensis*	Pinaceae	A	Megaphanerophyte	Mesohygrophyte	Cryophile	Acido-Neutrophilous
72	*Platanus × acerifolia*	Platanaceae	A	Megaphanerophyte	Mesohygrophyte	Mesothermal	Slightly Acido-Neutrophilous
73	*Phyllostachys aurea*	Poaceae	A	Mesophanerophyte	Hygrophyte	Moderate Thermophilic	Slightly Acido-Neutrophilous
74	*Frangula betulifolia*	Rhamnaceae	A	Mesophanerophyte	Mesophyte	Mesothermal	Slightly Acido-Neutrophilous
75	*Securinega suffruticosa*	Rhamnaceae	A	Nanophanerophyte	Xeromesophyte	Mesothermal	Slightly Acido-Neutrophilous
76	*Chaenomeles japonica*	Rosaceae	A	Nanophanerophyte	Mesophyte	Moderate Thermophilic	Slightly Acido-Neutrophilous
77	*Cotoneaster bullatus*	Rosaceae	A	Nanophanerophyte	Xeromesophyte	Mesothermal	Slightly Acido-Neutrophilous
78	*Cotoneaster melanocarpus*	Rosaceae	A	Nanophanerophyte	Xeromesophyte	Mesothermal	Slightly Acido-Neutrophilous
79	*Crataegus multiflora*	Rosaceae	A	Mesophanerophyte	Mesohygrophyte	Moderate Thermophilic	Neutro-Basophilous
80	*Crataegus phaenopyrum*	Rosaceae	A	Mesophanerophyte	Mesohygrophyte	Moderate Thermophilic	Neutro-Basophilous
81	*Cydonia oblonga*	Rosaceae	A	Mesophanerophyte	Mesophyte	Moderate Thermophilic	Slightly Acido-Neutrophilous
82	*Kerria japonica*	Rosaceae	A	Mesophanerophyte	Mesophyte	Mesothermal	Acido-Neutrophilous
83	*Malus domestica*	Rosaceae	A	Megaphanerophyte	Amphitolerant (Euryhydric)	Mesothermal	Slightly Acido-Neutrophilous
84	*Malus floribunda*	Rosaceae	A	Megaphanerophyte	Amphitolerant (Euryhydric)	Mesothermal	Slightly Acido-Neutrophilous
85	*Prunus domestica*	Rosaceae	A	Megaphanerophyte	Mesophyte	Mesothermal	Slightly Acido-Neutrophilous
86	*Prunus dulcis*	Rosaceae	A	Megaphanerophyte	Xerophyte	Moderate Thermophilic	Slightly Acido-Neutrophilous
87	*Prunus laurocerasus*	Rosaceae	A	Mesophanerophyte	Mesophyte	Moderate Thermophilic	Acido-Neutrophilous
88	*Prunus serrulata*	Rosaceae	A	Mesophanerophyte	Mesophyte	Moderate Thermophilic	Acido-Neutrophilous
89	*Prunus tomentosa*	Rosaceae	A	Mesophanerophyte	Mesophyte	Moderate Thermophilic	Acido-Neutrophilous
90	*Pyracantha coccinea*	Rosaceae	A	Mesophanerophyte	Xeromesophyte	Moderate Thermophilic	Slightly Acido-Neutrophilous
91	*Rhodotypos kerrioides*	Rosaceae	A	Nanophanerophyte	Mesophyte	Mesothermal	Acido-Neutrophilous
92	*Rosa rugosa*	Rosaceae	A	Nanophanerophyte	Xeromesophyte	Mesothermal	Acido-Neutrophilous
93	*Sorbaria sorbifolia*	Rosaceae	A	Nanophanerophyte	Mesohygrophyte	Mesothermal	Acido-Neutrophilous
94	*Spiraea bumalda*	Rosaceae	A	Nanophanerophyte	Mesohygrophyte	Mesothermal	Slightly Acido-Neutrophilous
95	*Spiraea × vanhouttei*	Rosaceae	A	Nanophanerophyte	Mesohygrophyte	Mesothermal	Slightly Acido-Neutrophilous
96	*Phellodendron amurense*	Rutaceae	A	Megaphanerophyte	Amphitolerant (Euryhydric)	Mesothermal	Acido-Neutrophilous
97	*Ptelea trifoliata*	Rutaceae	A	Mesophanerophyte	Mesophyte	Mesothermal	Slightly Acido-Neutrophilous
98	*Tetradium daniellii*	Rutaceae	A	Mesophanerophyte	Mesophyte	Moderate Thermophilic	Acido-Neutrophilous
99	*Tetradium ruticarpum*	Rutaceae	A	Mesophanerophyte	Mesophyte	Moderate Thermophilic	Acido-Neutrophilous
100	*Zanthoxylum piperitum*	Rutaceae	A	Nanophanerophyte	Xerophyte	Thermophilic	Slightly Acido-Neutrophilous
101	*Salix babylonica*	Salicaceae	A	Megaphanerophyte	Xeromesophyte	Mesothermal	Slightly Acido-Neutrophilous
102	*Salix matsudana*	Salicaceae	A	Megaphanerophyte	Xeromesophyte	Mesothermal	Neutro-Basophilous
103	*Acer ginnala*	Sapindaceae	A	Mesophanerophyte	Xeromesophyte	Mesothermal	Slightly Acido-Neutrophilous
104	*Acer laetum*	Sapindaceae	A	Megaphanerophyte	Xeromesophyte	Mesothermal	Slightly Acido-Neutrophilous
105	*Acer negundo*	Sapindaceae	A	Megaphanerophyte	Xeromesophyte	Mesothermal	Neutro-Basophilous
106	*Acer palmatum*	Sapindaceae	A	Mesophanerophyte	Xeromesophyte	Moderate Thermophilic	Slightly Acido-Neutrophilous
107	*Acer saccharinum*	Sapindaceae	A	Megaphanerophyte	Xeromesophyte	Mesothermal	Neutro-Basophilous
108	*Aesculus hippocastanum*	Sapindaceae	A	Megaphanerophyte	Mesohygrophyte	Moderate Thermophilic	Neutro-Basophilous
109	*Koelreuteria paniculata*	Sapindaceae	A	Megaphanerophyte	Xeromesophyte	Moderate Thermophilic	Neutro-Basophilous
110	*Xanthoceras sorbifolium*	Sapindaceae	A	Mesophanerophyte	Mesophyte	Moderate Thermophilic	Neutro-Basophilous
111	*Buddleja davidii*	Scrophulariaceae	A	Nanophanerophyte	Mesophyte	Moderate Thermophilic	Neutro-Basophilous
112	*Lycium halimifolium*	Solanaceae	A	Nanophanerophyte	Xerophyte	Mesothermal	Neutro-Basophilous
113	*Taxodium distichum*	Taxodiaceae	G	Megaphanerophyte	Hygrophyte	Mesothermal	Acido-Neutrophilous
114	*Taxus baccata* var. *globosa*	Taxaceae	G	Nanophanerophyte	Xeromesophyte	Mesothermal	Acido-Neutrophilous
115	*Celtis occidentalis*	Ulmaceae	A	Megaphanerophyte	Amphitolerant (Euryhydric)	Moderate Thermophilic	Neutro-Basophilous
116	*Viburnum burejaeticum*	Viburnaceae	A	Nanophanerophyte	Mesophyte	Mesothermal	Slightly Acido-Neutrophilous
117	*Viburnum carlesii*	Viburnaceae	A	Nanophanerophyte	Mesophyte	Mesothermal	Slightly Acido-Neutrophilous
118	*Viburnum orientale*	Viburnaceae	A	Mesophanerophyte	Mesophyte	Mesothermal	Slightly Acido-Neutrophilous
119	*Viburnum rhytidophyllum*	Viburnaceae	A	Mesophanerophyte	Mesophyte	Mesothermal	Slightly Acido-Neutrophilous
120	*Wisteria sinensis*	Wisteriaceae	A	Megaphanerophyte	Mesophyte	Moderate Thermophilic	Acido-Neutrophilous

* A = Angiosperm; G = Gymnosperm.

**Table 3:** The significance of the ecological requirements of plant species for the factors moisture, temperature, and soil pH [73,74].

Ecological Preference Index	Ecological Significance Description for Moisture	Ecological Significance Description for Temperature	Ecological Significance Description for Soil pH
0	Amphitolerant (Euryhydric)	Amphitolerant (Eurythermal)	Amphitolerant (Euryionic)
1–1.5	Xerophyte	Cryophile	Strongly Acidophilous
2–2.5	Xeromesophyte	Microthermal	Acidophilous
3–3.5	Mesophyte	Mesothermal	Acido-Neutrophilous
4–4.5	Mesohygrophyte	Moderately Thermophilic	Slightly Acido-Neutrophilous
5–5.5	Hygrophyte	Thermophilic	Neutro-Basophilous
6	Hydrophyte	-	-

## Data Availability

The data supporting the findings of the study are available within the article.

## References

[1] Dümpelmann S. (2024). Tree Times: Urban Plants as Timekeepers and Seasonal Indicators. J. Urban Hist..

[2] Simovic M., Mueller K.E., McMahon S.M., Medeiros J.S. (2024). Functional traits and size interact to influence growth and carbon sequestration among trees in urban greenspaces. Funct. Ecol..

[3] Niu C., Shou W., Ma L., Qian J. (2022). Tree height-related hydraulic strategy to cope with freeze-thaw stress in six common urban tree species in North China. Phyton-Int. J. Exp. Bot..

[4] Lahoti S., Lahoti A., Joshi R.K., Saito O. (2020). Vegetation structure, species composition, and carbon sink potential of urban green spaces in Nagpur City, India. Land.

[5] Heneidy S.Z., Halmy M.W.A., Toto S.M., Hamouda S.K., Fakhry A.M., Bidak L.M., Eid E.M., Al-Sodany Y.M. (2021). Pattern of Urban Flora in Intra-City Railway Habitats (Alexandria, Egypt): A Conservation Perspective. Biology.

[6] Hirons A.D., Watkins J.H.R., Baxter T.J., Miesbauer J.W., Male-Muñoz A., Martin K.W.E., Bassuk N.L., Sjöman H. (2021). Using botanic gardens and arboreta to help identify urban trees for the future. Plants People Planet.

[7] Horvat E., Sipek M., Sajna N. (2024). Urban hedges facilitate spontaneous woody plants. Urban For. Urban Green..

[8] Yang J., Cen C., Wang Z., Jian M. (2024). Impacts of spatiotemporal urban expansion on the species richness and functional traits of adults and sapling woody trees and shrubs of urban remnant forest patches. Ecol. Indic..

[9] Muhlisin J.I., Gunawan B., Cahyandito M.F. (2021). Vegetation diversity and structure of urban parks in Cilegon City, Indonesia, and local residents’ perception of its function. Biodiversitas.

[10] Bartoli F., Savo V., Caneva G. (2022). Biodiversity of urban street trees in Italian cities: A comparative analysis. Plant Biosyst..

[11] Nero B., Kuusaana E., Ahmed A., Campion B. (2024). Carbon storage and tree species diversity of urban parks in Kumasi, Ghana. City Environ. Interact..

[12] Fonseca W., Martini A., Martins S., Oliveira M., Duenez L., Alves W. (2024). Exploring urban forests in Minas Gerais, Brazil: Floristic diversity and biome-driven insights to green infrastructure planning. Urban Ecosyst..

[13] Muvengwi J., Ndagurwa H., Witkowski E., Mbiba M. (2024). Woody species composition, diversity, and ecosystem services of yards along an urban socioeconomic gradient. Sci. Total Environ..

[14] Jang J., Woo S. (2022). Native Trees as a Provider of Vital Urban Ecosystem Services in Urbanizing New Zealand: Status Quo, Challenges and Prospects. Land.

[15] Galfrascoli G., Bernardello G., Calviño A. (2023). How well do trees fit the city? Lessons from an urban tree survey in Córdoba, Argentina. Bol. Soc. Argent. Bot..

[16] Rogovskyi S., Ishchuk L., Ishchuk H. (2023). Chornobyl’s current dendroflora: Analysis of natural successions in the abandoned urban phytocoenoses. Trak. Univ. J. Nat. Sci..

[17] Postarnak Y., Zhavoronkov V. (2023). Urban dendroflora of dry subtropics of the northwestern part of the greater Caucasus on the example of the city of Gelendzhik. Russ. J. Earth Sci..

[18] Lakicevic M., Reynolds K.M., Orlovic S., Kolarov R. (2022). Measuring dendrofloristic diversity in urban parks in Novi Sad (Serbia). Trees For. People.

[19] Seboko T., Shackleton C., Ruwanza S. (2024). Urban residents’ knowledge of and attitudes and willingness to control woody invasive alien plants in their domestic gardens in South Africa. People Nat..

[20] Nielsen A.B., van den Bosch M., Maruthaveeran S., Konijnendijk van den Bosch C. (2014). Species richness in urban parks and its drivers: A review of empirical evidence. Urban Ecosyst..

[21] Nobre Lisboa M.A., Alves da Silva L.V., da Silva Nascimento A., de Oliveira Silva A., Alves Teixeira M.R., Ferreira M.F.R., Cardoso Fereira S., Vieira da Silva A.C., Viana Corales A., Tovares Calixto J. (2024). Diversity, structure, and carbon sequestration potential of the woody flora of urban squares in the Brazilian semiarid region. Trees For. People.

[22] Dimitrova A., Stipanovic V., Kolevska D. (2023). Collection of Experiences: 25 Years’ Work on Seed Propagation of Allochthonous Woody Plants in Skopje and Their Possible Role in the Urban Landscape. Seefor-South-East Eur. For..

[23] Palaj A., Kollár J. (2021). Expansion of phanerophytes above the timberline in the Western Carpathians. Biologia.

[24] Stehlik I., Caspersen J., Wirth L., Holderegger R. (2007). Floral free fall in the Swiss lowlands: Environmental determinants of local plant extinction in a peri-urban landscape. J. Ecol..

[25] Yang J., Wang Z., Pan Y., Zheng Y. (2023). Woody plant functional traits and phylogenetic signals correlate with urbanization in remnant forest patches. Ecol. Evol..

[26] Stagoll K., Lindenmayer D.B., Knight E., Fischer J., Manning A.D. (2012). Large trees are keystone structures in urban parks. Conserv. Lett..

[27] Campbell-Arvai V., Vergel R., Lindquist M., Fox N., Van Berkel D. (2024). Tree selection for a virtual urban park: Comparing aided and unaided decision-making to support public engagement in greenspace design. Urban For. Urban Green..

[28] Fini A., Vigevani I., Corsini D., Wężyk P., Bajorek-Zydron K., Failla O., Cagnolati E., Mielczarek L., Comin S., Gibin M. (2024). CO_2_ assimilation, sequestration, and storage by urban woody species growing in parks and along streets in two climatic zones. Sci. Total Environ..

[29] Salinitro M., Alessandrini A., Zappi A., Melucci D., Tassoni A. (2018). Floristic diversity in different urban ecological niches of a southern European city. Sci. Rep..

[30] Lin B.B., Ossola A., Ripple W., Alberti M., Andersson E., Bai X., Dobbs C., Elmqvist T., Evans K., Frantzeskaki N. (2021). Integrating solutions to adapt cities for climate change. Lancet Planet. Health.

[31] Morozkin A., Kalimullina S., Salova L., Shpak T. (2001). Status of forest ecosystems in the impact zone of the Nizhnekamsk industrial complex. Eurasian Soil Sci..

[32] Liu Y., Duan X., Li X., Yi W., Chen G., Yang J., Deng D., Guo X., Yang Z., Huang G. (2024). Anti-seasonal flooding drives substantial alterations in riparian plant diversity and niche characteristics in a unique hydro-fluctuation zone. Ecol. Evol..

[33] Géron C., Lembrechts J., Nijs I., Monty A. (2022). Woody invaders from contrasted climatic origins distribute differently across the urban-to-rural gradient in oceanic Europe—Is it trait-related?. Urban For. Urban Green..

[34] Yang S., Wang L., Stathopoulos T., Marey A.M. (2023). Urban microclimate and its impact on built environment-A review. Build. Environ..

[35] You W., Liang Y. (2024). Numerical investigation of different building configurations for improving outdoor spatial ventilation conditions in strip-type residential neighbourhoods. Urban Clim..

[36] Li S., Zhu Y., Wan H., Xiao Q., Teng M., Xu W., Qiu X., Wu X., Wu C. (2024). Effectiveness of potential strategies to mitigate surface urban heat island: A comprehensive investigation using high-resolution thermal observations from an unmanned aerial vehicle. Sustain. Cities Soc..

[37] Teodorescu T., Guidi W., Labrecque M. (2011). The use of non-dormant rods as planting material: A new approach to establishing willow for environmental applications. Ecol. Eng..

[38] Jensen J., Holm P., Nejrup J., Larsen M., Borggaard O. (2009). The potential of willow for remediation of heavy metal polluted calcareous urban soils. Environ. Pollut..

[39] Grignet A., de Vaufleury A., Papin A., Bert V. (2020). Urban soil phytomanagement for Zn and Cd in situ removal, greening, and Zn-rich biomass production taking care of snail exposure. Environ. Sci. Pollut. Res..

[40] Fan L., Wang J., Han D., Gao J., Yao Y. (2023). Research on Promoting Carbon Sequestration of Urban Green Space Distribution Characteristics and Planting Design Models in Xi’an. Sustainability.

[41] Andronova M., Platonov A. (2022). Sucrose in the tissues of annual shoots of introduced woody plants. Lesn. Zhurnal-For. J..

[42] Lipatov D., Varachenkov V., Manakhov D., Mamikhin S., Shcheglov A. (2023). ^137^Cs pollution in soils and plants of urban ecosystems near the Elektrostal Heavy Machinery Plant. Biol. Bull..

[43] Muhammad S., Wuyts K., Samson R. (2022). Selection of plant species for particulate matter removal in urban environments by considering multiple ecosystem (dis)services and environmental suitability. Atmosphere.

[44] Hostyn G., Schwartz C., Côme J., Ouvrard S. (2022). Assessment for combined phytoremediation and biomass production on a moderately contaminated soil. Environ. Sci. Pollut. Res..

[45] Hamberg L., Lehvävirta S., Kotze D., Heikkinen J. (2015). Tree species composition affects the abundance of rowan *Sorbus aucuparia* L.) in urban forests in Finland. J. Environ. Manag..

[46] Ridgway K.P., Marland L.A., Harrison A.F., Wright J., Young J.P.W., Fitter A.H. (2004). Molecular diversity of *Frankia* in root nodules of *Alnus incana* grown with inoculums from polluted urban soils. FEMS Microbiol. Ecol..

[47] Krisans O., Caksa L., Matisons R., Rust S., Elferts D., Seipulis A., Jansons A. (2022). A static pulling test is a suitable method for comparison of the loading resistance of silver birch *Betula pendula* Roth.) between urban and peri-urban forests. Forests.

[48] Krisans O., Matisons R., Kitenberga M., Donis J., Rust S., Elferts D., Jansons A. (2021). Wind resistance of Eastern Baltic silver birch (*Betula pendula* Roth.) suggests its suitability for periodically waterlogged sites. Forests.

[49] Petrushkevych Y., Korshykov I. (2020). Ecological and biological characteristics of *Betula pendula* in the conditions of urban environment. Regul. Mech. Biosyst..

[50] Nouri K., Nikbakht A., Haghighi M., Etemadi N., Rahimmalek M., Szumny A. (2023). Screening some pine species from North America and dried zones of western Asia for drought stress tolerance in terms of nutrients status, biochemical and physiological characteristics. Front. Plant Sci..

[51] Kalberer S.R., Wisniewski M., Arora R. (2006). Deacclimation and reacclimation of cold-hardy plants: Current understanding and emerging concepts. Plant Sci..

[52] Chew Y.H., Wilczek A.M., Williams M., Welch S.M., Schmitt J., Halliday K.J. (2012). An augmented *Arabidopsis* phenology model reveals seasonal temperature control of flowering time. New Phytol..

[53] Klisz M., Puchalka R., Netsvetov M., Prokopuk Y., Vítková M., Sádlo J., Matisons R., Mionskowski M., Chakraborty D., Olszewski P. (2021). Variability in climate-growth reaction of *Robinia pseudoacacia* in Eastern Europe indicates potential for acclimatisation to future climate. For. Ecol. Manag..

[54] Esperon-Rodriguez M., Power S., Tjoelker M., Marchin R., Rymer P. (2021). Contrasting heat tolerance of urban trees to extreme temperatures during heatwaves. Urban For. Urban Green..

[55] Wright A., Francia R. (2024). Plant traits, microclimate temperature and humidity: A research agenda for advancing nature-based solutions to a warming and drying climate. J. Ecol..

[56] Maes S.L., Perring M.P., Depauw L., Bernhardt-Römermann M., Blondeel H., Brūmelis G., Brunet J., Decocq G., den Ouden J., Govaert S. (2020). Plant functional trait response to environmental drivers across European temperate forest understorey communities. Plant Biol..

[57] Song G., Wang J., Han T., Wang Q., Ren H., Zhu H., Wen X., Hui D. (2019). Changes in plant functional traits and their relationships with environmental factors along an urban-rural gradient in Guangzhou, China. Ecol. Indic..

[58] Marcenò C., Guarino R. (2015). A test on Ellenberg indicator values in the Mediterranean evergreen woods *Quercetea ilicis*. Rend. Lincei-Sci. Fis. E Nat..

[59] Lososová Z., Chytrý M., Cimalová S., Kropáč Z., Otýpková Z., Pyšek P., Tichý L. (2004). Weed vegetation of arable land in Central Europe: Gradients of diversity and species composition. J. Veg. Sci..

[60] Di Biase L., Tsafack N., Pace L., Fattorini S. (2023). Ellenberg indicator values disclose complex environmental filtering processes in plant communities along an elevational gradient. Biology.

[61] Lawesson J.E. (2003). pH optima for Danish forest species compared with Ellenberg reaction values. Folia Geobot..

[62] Carpenter W., Goodenough A. (2014). How robust are community-based plant bioindicators? Empirical testing of the relationshipbetween Ellenberg values and direct environmental measures in woodland communities. Community Ecol..

[63] Zalacáin D., Martínez-Pérez S., Bienes R., García-Díaz A., Sastre-Merlín A. (2019). Salt accumulation in soils and plants under reclaimed water irrigation in urban parks of Madrid (Spain). Agric. Water Manag..

[64] Dmuchowski W., Baczewska-Dabrowska A., Gozdowski D., Bragoszewska P., Gworek B., Suwara I., Chojnacki T., Jóźwiak A., Swiezewska E. (2021). Effect of salt stress in urban conditions on two *Acer* species with different sensitivity. PEERJ.

[65] Rahmonov O., Kowal A., Rahmonov M., Pytel S. (2024). Variability of concentrations of potentially toxic metals in the topsoil of urban forest parks (Southern Poland). Forests.

[66] Chytrý M., Tichý L., Roleček J. (2002). Local and regional patterns of species richness in Central European vegetation types along the pH/calcium gradient. Folia Geobot..

[67] Ciupa V. (2018). Timișoara—Oraș Grădină, oraș al Parcurilor, Oraș al Florilor—Monografie.

[68] Ciupa V. (2010). Cadrul Natural şi Peisagistic al Municipiului Timişoara. Volumul I. https://hcl.civicul.ro/view-hcl/hcl_124_30.03.2010/attachment/001_cadrul_natural_vol.1.pdf.

[69] Primăria Timișoara—Registrul Local al Spațiilor Verzi—Municipiul Timișoara—Parcul Botanic, Executant S.C. Geotop S.R.L., Beneficiar Primăria Timișoara, Faza “Actualizarea Lucrării «Cadastru Verde Existent (Parcuri) pe Baza Datelor Existente la Beneficiar»”, No Document Datation.

[70] Raunkiaer C. (1934). The Life Forms of Plants and Statistical Plant Geography.

[71] Sârbu A., Smarandache D., Pascale G. (2003). Îndrumător de Practică Botanică: Munţii Bucegi-Baiului.

[72] Cristea V., Gafta D., Pedrotti F. (2004). Fitosociologie.

[73] Sanda V., Popescu A., Doltu M.I., Doniţă N. (1983). Caracterizarea Ecologică şi Fitocenologică a Speciilor Spontane din Flora României.

[74] Sanda V., Stefanut S. (2003). Atlas Florae Romaniae. I. Pinophytina.

[75] Ciocârlan V. (2000). Flora Ilustrată a României.

[76] Ellenberg H. (1952). Landwirtschaftliche Pflanzensoziologie II. Wiesen und Weiden und ihre Standörtliche Bewertung.

